# The Psychology of Addictive Smartphone Behavior in Young Adults: Problematic Use, Social Anxiety, and Depressive Stress

**DOI:** 10.3389/fpsyt.2020.573473

**Published:** 2020-09-15

**Authors:** Aurel Pera

**Affiliations:** Department of Teacher Training, University of Craiova, Craiova, Romania

**Keywords:** smartphone, behavior, anxiety, depression, stress

## Abstract

This review enhances the existing literature on relationships between problematic smartphone use (PSU), psychopathology, addictive personality, and online social engagement as regards young adults, giving attention to predictive determinants of addictive behavior in smartphone usage. My article cumulates previous research findings on the psychology of addictive smartphone behavior in terms of problematic use, social anxiety, and depressive stress by focusing on the relationship among mobile social media usage, smartphone addiction risk, mental health issues, and individual well-being. The inspected collected findings prove that depression and social anxiety constitute risk determinants for greater PSU and that particular categories of smartphone applications are positively related to well-being. State anxiety and motivations represent significant predictors of PSU. High PSU affects participation in social engagement. As limitations in the current review, my results point towards relevant avenues of research on social consequences of teenagers’ smartphone problematic use. Future directions should clarify whether compulsive smartphone use adversely affects both mental and physical health in the long run.

## Introduction

Problematic smartphone use (PSU) is a complex phenomenon comprising diverse dysfunctional manifestations (*e.g.* social isolation, diminished self-confidence, depression, and anxiety) without being a potential behavioral addiction (1). Smartphones may have negative consequences on the human brain and associated psychological processes. Based on psychiatric symptoms, various kinds of smartphone use disorder have been identified, some of them associated with Internet communication, particularly as PSU is related to depression and self-esteem (2). I will extend the argument by positing that as PSU is negatively related to young adults’ psychosocial well-being, time spent using smartphones influences mental health. I initially undertook a review of Web of Science in November 2019, supplemented with updates from this database and from Scopus and ProQuest in May 2020. Search terms included “smartphone addiction”, “smartphone addiction risk”, “smartphone addiction predisposition”, “addictive smartphone behavior”, “problematic smartphone use”, and “compulsive smartphone use”. As I focused on journal articles published between 2017 and 2020, excluding editorial material, only 267 original research and review articles met the eligibility criteria. By removing those whose results were inconclusive, unconfirmed by replication or too general, and because of space constraints, I selected only 67 articles (**Table 1**). My review provides evidence that PSU is adversely associated with diverse notions of well-being and that symptoms of psychopathology are related to severity of PSU, with flimsy evidence though that smartphone use can be addictive as drug and alcohol use disorders are.

**Table 1:** Topics and types of papers identified and selected.

Topic	Identified	Selected
smartphone addiction	102	52
smartphone addiction risk	44	17
smartphone addiction predisposition	42	14
addictive smartphone behavior	60	26
problematic smartphone use	121	54
compulsive smartphone use	69	22
**Type of paper**		
original research	241	63
review	26	4

Source: Processed by the author. Some topics overlap.

## PSU and Depression Psychopathology

PSU is sometimes positively associated with adolescent depression. As an illustration, perceived social support moderates the relationship between procrastination and adolescent depression, being relevant only for teenagers having a low degree of perceived social support (3). For such teenagers, the connection between sensation seeking and smartphone use is positively relevant, while for teenagers having a significant degree of perceived social support, the link between sensation seeking and PSU is strong (4). PSU, stress degrees, and perceived social support are more significant among women students (5). Self-command, apprehension, depression, and dysfunctional impulsivities are the most significant predictors of PSU (6), and certain groups of patients having schizophrenia may necessitate special care to hinder PSU (7). Unsociable persons may depend on smartphone wishing to be connected with and obtain support from other users, while possibly leading up to confronting PSU (8).

Difficulties appear when users are addicted to their smartphone, spending an excessive quantity of time using it. Dysfunctional symptoms of PSU mirror substance use disorder criteria (9–11). An immoderate utilization of smartphones leads to symptomatology reminiscent of psychological disorders generated by substance addiction (12). Smartphones are memory extenders, functioning as convenient data depository from the ordinary to the intricate (13).

PSU has public health relevance (14) because of irrefutable links with alcohol use, particular mental health diagnoses (*e.g.* depression, anxiety, ADHD, PTSD, *etc.*), and inferior academic performance (15). Depressive disorders mediate the link between academic stress and PSU. Individuals having lower or moderate degrees of problem-focused coping are likely to display more depressive symptoms, generating a higher score on PSU (16). The adverse association between smartphone use and academic performance may result in an addictive behavior that may affect students’ careers (17).

PSU is associated with anxiety symptom severity and depression psychopathology, shaping personality factors, self-esteem, and perceived social support. Psychological distress, addictive personality, and emotion dysregulation are instrumental in configuring PSU severity.

## Cognitive Behavioral Processes and PSU

The effect of cognitive absorption on PSU is mediated by addiction to social media. PSU is higher than social media excessive use (18) and differs by educational expertise, while social media practice is not dissimilar by gender, age, or instruction. Investigating users’ degrees of cognitive absorption (their state of participation in software and technology) with PSU may be instrumental in grasping the influence of user experiences in computer-mediated settings. Individuals dependent on smartphones and social media undergo significant degrees of cognitive absorption (19), especially by women when using social media and higher for social media than smartphones. Therefore, users reporting PSU communicate more significant degrees of cognitive absorption than those disclosing inferior degrees of addiction (20), while users having low degrees of PSU display more significant levels of cognitive absorption than those having no degree of PSU. Grasping user experiences or opinions impact user behavior (21), and clarifies how users become immersed in technology. The impact of user experiences and the association with PSU involves how individuals participate in technology (22) and may become thoroughly absorbed in it, in some cases to a troublesome extent (23).

Smartphone application personalities such as collaborative and ambitious, captivating and appealing, communicative and rigorous, and conscientious and righteous all indicate the robust interactive attribute of such devices. Smartphone users’ behaviors, smartphone social applications that link individuals, and perceived social capital resulted *via* smartphone social application practice drive smartphone application personality. Besides having hedonic value, leisure applications can be instrumental in social capital in addition to the smartphone application personality aspects (24). Context-awareness is assimilable into the system to adequately detect a smartphone user taking into account the corresponding information. Smartphones are increasingly cognitive and context-aware (25) with developing perceiving, interconnection, and computing capabilities. As soon as a user is located, his/her activities can be monitored for health supervision (26), social networking scrutiny, and prediction and modeling of way of living. Ceaseless authentication of smartphone users can be inspected in conformity with their behavioral features employing activity pattern identification (27). Because of this, functional dependence of PSU draws attention to instrumental practicality of the smartphone (functionally dependent users tend to be more inclined to change PSU), while existential dependence of PSU addresses compulsive, frequently unconscious, attachment (existential dependent users tend to be more disinclined in acknowledging adverse consequences) (28).

Behavioral addiction and pathological personality traits clarify depression and anxiety’s associations with PSU-related dysfunctional mechanisms and motivational processes, impacting subjective and psychological well-being.

## Addictive Smartphone Behavior and Physical Health

Physical negative effects are rooted in excessive smartphone use (29). However, addictive smartphone behavior may resemble addiction as regards immoderate use (30), impulse control issues, and adverse effects, without being a disorder with serious consequences on physical and psychological health (31). For example, smartphone texting is associated with a more immobile and more tense spinal position in contrast to desktop computer typing (32). Persistent neck flexion throughout smartphone represents a determinant of neck discomfort and modification of neck muscle performance. Shoulder taping diminishes neck pain without impacting neck muscle performance and tiredness during smartphone texting (33).

Excessive smartphone use also generates poor mental health outcomes, *e.g.* depressive symptoms and sleep issues (34). Concerning smartphone and social platform adoption, positive results are associated with social capital and involvement (35), while detrimental consequences develop out of excessive use, negative correlations, and the anxiety of being perpetually active. As an illustration, not employing smartphones in the bedroom boosts the standard of living, and risk of smartphone overuse diminishes when such devices are kept outside the bedroom. Sleeping without smartphones enhances sleep quality, relationships, motivation, and good physical condition (36). PSU can bring about mobility issues in the wrists, fingers and neck, in addition to intrusion into sleep habits. For teenagers, the quality of sleep impacts growth, emotional constancy, and learning abilities, and thus the handling of PSU is decisive for adequate sleeping habits (37).

Higher sensation seeking persons may be particularly at risk of PSU. Smartphones may enable individuals to be involved in a range of undertakings (38) and assist them in alleviating leisure boredom. More significant levels of leisure boredom and sensation seeking (39) have a positive link with a higher degree of smartphone overuse. Smartphones may be addictive, and their users aiming to attain various purposes can become extremely dependent on them. Individuals having stronger and more straightforward grounds to attempt to accomplish objectives on their smartphones (40) are more predisposed to have a more significant level of smartphone overuse (41).

Excessive smartphone use may lead to addictive smartphone behavior that may result in physical and psychological negative effects, impacting, among others, neck muscle performance and sleep quality.

## Smartphone Use and Social Anxiety

There is a moderate link between smartphone use and stress and anxiety (42), but depression severity and anxiety severity are significantly associated with excessive smartphone use (43). State anxiety and motivations represent significant predictors of PSU. State anxiety moderates the link between leisure and social interaction motivations and PSU for the high smartphone-use group, while not moderating such relationship for the low smartphone-use group (44). Bringing together various functions, smartphones supply teenagers with multiple entertainment prospects to alleviate leisure boredom. Smartphone adoption for both interpersonal networking and relaxation (45) has important and direct consequences on smartphone overuse (46), while individuals who use smartphones for news pursuit are not affected by PSU. Incapacity to regulate addiction, escape, anxiety, and productivity loss all constitute a variety of smartphone users’ behavioral effects from PSU (41). Significant alienation can escalate PSU (47).

Despite the fact that the compulsive nature of smartphone users is less relevant than extraversion in giving rise to smartphone social application practice, smartphone application personality, and social capital constitution, such devices are beneficial for persons high in anxiety to obtain social capital. The self-observing and selectiveness characteristics of bonding social capital drive it to be negatively instrumental in the collaborative and appealing smartphone application personality (24). Thus, depression and social anxiety constitute risk determinants for greater PSU. Specifically, body image dissatisfaction is a positive predictor of PSU. Social anxiety is a relevant mediator between body image dissatisfaction and PSU. Along with it, for socially anxious persons, smartphone-based online networking is less nerve-racking than offline social one. Emotionally traumatic experiences are related to PSU in teenagers and this link may be somewhat clarified by body image dissatisfaction and psychosocial risk determinants (48).

Depression and social anxiety are correlates of PSU. Psychopathological symptoms, interpersonal sensitivity, and smartphone use articulate the behavioral dynamics of social anxiety symptom severity.

## Stress, Subjective and Psychological Well-Being, and PSU

PSU is adversely associated with diverse notions of well-being, *e.g.* subjective contentment, mental health, and depression or stress disorders. Adverse affect, self-determination, and environmental proficiency have negative associations with PSU. Well-being and accompanying dispositional determinants may bring about perceived and actual PSU. Considering the steady and dispositional character of well-being (49), such links are regulated by a shared intrinsic predisposition to experience anxiety, deleterious emotions, and an absence of control, integrated into a propensity to get involved in dysfunctional coping and compulsive behavior (50). Depression, anxiety, and self-control are thus risk determinants or results of PSU that is also compulsive, typified by immoderate time spent on smartphones, invasion of social networking and responsibilities, and stress in detaching from such devices (51). Perceived stress is positively associated with adolescent smartphone overuse. Self-regulation mediates the association between perceived stress and smartphone addiction. The direct and indirect consequences are moderated by mindfulness, being more relevant for persons with inferior degrees of mindfulness (52).

Total application screen time is related to relationship conflict, in addition to anxiety and depression. The adoption of smartphones to interact with friends causes emotional states of deception, remorse, and feeling stressed out to respond promptly to their smartphone communications. Increased smartphone adoption results in problematic use (19) as the outward world effortlessly permeates users’ private lives. Smartphones enable the adjustability and non-confinement to collect information, have fun, and interact, with few impediments, while users are also under compulsion to their mobile devices. Particular categories of smartphone applications are positively related to well-being, but the predisposition for incessant connection has generated a range of problems associated with PSU (53). Smartphones may be instrumental in sharing any instinctive reflection or experience with users’ social networks. Egocentric teenagers’ validation-seeking on social media, especially as a manner to surmount social exclusion (54), may have unwelcome repercussions (38) and eventually results in a developing model of counterproductive behavior. Earlier teenage egocentrism predicts later social media disclosure, PSU, and interactional stress, through increased validation-seeking. Smartphones are equipped to boost teenagers’ persistent utilization of social media (49) for intentions of assertiveness, interpersonal relationship, and social attention (55), thus generating higher degrees of stress and dependency associated with such devices (56).

Both process- and social-led smartphone characteristic uses are associated with greater PSU (that is excessive utilization of smartphones increases risk of PSU) (57). PSU has certain patterns depending on age, with preponderance of authority, mood alteration, and discord in prepubescence, and patience, withdrawal symptoms, and indisposition in pubescence (58). For example, grandiose narcissism is positively related to PSU, while the consequence of vulnerable narcissism is entirely mediated by disinterest (59). PSU also can deteriorate teenage self-esteem, procrastination mediating the association between them (60), and more, teenagers are prone to addiction by lacking the strength to regulate impulsive behavior (61). Students having a relationship make more significant use of smartphones than those who are partnerless (62).

PSU shapes subjective and psychological well-being, and may configure associated anxiety and depression psychopathology. Mental health and somatic symptoms are adversely impacted by PSU leading to impaired socio-emotional functioning and possibly causing psychological distress.

## Parental Neglect and PSU

Users’ psychological features may not clarify every characteristic of PSU. High PSU affects participation in social engagement. Users’ absence of social networks may hinder agreeable social communications (49) and emotional states of encouragement in the offline setting, which may intensify their intention to escape to smartphones. As persons having PSU pursue more straightforward interactions than those undergone in the concrete realm (63), smartphones may have distinct consequences on their social ways of life than other users’. Formal organizational involvement, level of interaction with parents, dimension of the peer group, and fellow support diminish PSU (64). Depression moderates the association between sensation seeking and adolescent PSU. Sensation seeking is positively related to adolescent smartphone problematic use. For teenagers having an inferior degree of depression, the link between sensation seeking and PSU is positively relevant, while for teenagers having a significant degree of depression, the connection between sensation seeking and PSU is not relevant (4).

Parental neglect is considerably related to teenagers’ smartphone addiction. In the link between parental neglect and PSU, the former is not relevantly connected with the relational instability with peers, negatively shaping PSU. The relational instability with teachers has a fragmentary mediation impact between parental neglect and PSU (65). Parents who have significant degrees of risk perception and mediation performance are more predisposed to carry out restrictive mediation of the Internet and mobile devices use by their children. Children who have unsatisfactory academic performance, depression, possess smartphones, frequently play tablet gaming, and routinely use social media and instant messaging have inferior degrees of parental restrictive mediation and of acknowledged online safety knowledge, while being more inclined to experience PSU (66). Interpersonal adaptation mediates the link between parental attachment and PSU, and is moderated by self-control, being more intense for persons having lower self-control (67).

Parenting style and attachment may mediate young adults’ smartphone use, improving interpersonal adaptation and self-control, while articulating family well-being. Young adults’ addictive personality may be shaped by parental mediation practices and self-regulation.

## Conclusions

Significant research has considered lately the psychology of addictive smartphone behavior in terms of problematic use, social anxiety, and depressive stress. My article extends previous work by focusing on the relationship among mobile social media usage, smartphone addiction risk, mental health issues, and individual well-being. Progressively relevant degrees of smartphone ownership and utilization give rise to the implicit risk for addictive behaviors and adverse health results, shaping subjective and psychological well-being. The conclusions drawn from the above analyses are that depression and anxiety symptoms are associated with PSU severity, generating, among others, emotion dysregulation, psychological distress, poor sleep quality, and diminished academic performance. Personality traits, social-emotional distress, and duration of daily smartphone use constitute antecedents of PSU, impacting subjective and psychological well-being. As limitations in the current review, my results point towards relevant avenues of research on social consequences of young adults’ smartphone addiction. Future directions should clarify whether compulsive smartphone use adversely affects both mental and physical health in the long run.

## Author Contributions

The author confirms being the sole contributor of this work and approved it for publication.

## Conflict of Interest

The author declares that the research was conducted in the absence of any commercial or financial relationships that could be construed as a potential conflict of interest.
